# Supplementary material for: Modelling the ultra-strongly coupled spin-boson model with unphysical modes
Source: Nat Commun. 2019 Aug 19;10:3721. doi: 10.1038/s41467-019-11656-1 (PMC6700178; doi:10.1038/s41467-019-11656-1)
Supplement: Supplementary file 1 — Supplementary Information [file 41467_2019_11656_MOESM1_ESM.pdf]

Supplemental Information:
Modelling the ultra-strongly coupled spin-boson model with unphysical modes

Neill Lambert, Shahnawaz Ahmed, Mauro Cirio, and Franco Nori

Supplementary Note 1: Reaction coordinate (RC) mapping

The reaction coordinate (RC) mapping is described in detail in Refs. [1–4], and we will only discuss it briefly. After the mapping, one can derive an appropriate master equation description of the residual bath. For the full RC-model to which we compare the hierarchical equations of motion (HEOM) results in the main paper we use a Born-Markov-secular master equation description of the residual bath [described by the d_k modes in Eq. (16) in the main text], which has the form

$$\dot{\rho} = -i[H_{\mathrm{RC}}, \rho] + D^{(1)}[\rho] \ , \tag{1}$$

where

$$H_{\mathrm{RC}} = \frac{\omega_{\mathrm{q}}}{2}\sigma_z + \frac{\Delta}{2}\sigma_x + \sigma_z \frac{\lambda}{\sqrt{2\omega_0}}(a + a^\dagger) + \omega_0 a^\dagger a \ , \tag{2}$$

and

$$D^{(1)}[\rho] = \sum_{i,j>i} D_{i,j}[\rho] \tag{3}$$

$$D^{(1)}_{i,j}[\rho] = J_{\mathrm{res}}(\Delta_{i,j})\bar{X}_{i,j}\,[2|\psi_i\rangle\langle\psi_j|\rho|\psi_j\rangle\langle\psi_i| - |\psi_j\rangle\langle\psi_j|\rho - \rho|\psi_j\rangle\langle\psi_j|] \, .$$

Here $\Delta_{i,j}$ is the energy difference between the eigenstates ψ_i and ψ_j of H_{RC}. In addition, $\bar{X}_{i,j} = |\langle\psi_j|\hat{X}|\psi_i\rangle|^2$, and $\hat{X} = (a + a^\dagger)/\sqrt{2\omega_0}$.

This master equation, Supplementary Eq. (1), is used to produce the purple dashed curves in Figures 3 and 4 in the main text. For small values of γ (narrow spectral densities) this qualitatively approximates the HEOM result. The master equation predicts an energy dissipation into the environment in the following form

$$J(t) = \sum_{i,j>i} \Delta_{i,j} J_{\mathrm{res}}(\Delta_{i,j})\bar{X}_{i,j}\,\mathrm{Tr}\left(|\psi_j\rangle\langle\psi_j|\rho(t)\right) . \tag{4}$$

RC with RWA and flat-bath spectral density

We wish to see the effect of removing the Matsubara terms from the RC method. In the HEOM method, it is as straightforward as ignoring them in the correlation function. In the main text we saw that, via a comparison to the pseudomode result, it was clear that for the RC model neglecting the Matsubara frequencies was equivalent to making a series of approximations on the residual environment.

It is also useful to arrive at that same conclusion with a different argument, following the discussion by Ingold[5]. To start, we rewrite the spectral density in Eq. (3) in the main text as a sum of two Lorentzians

$$J(\omega) = \frac{\gamma\lambda^2}{4\Omega}\left[\frac{1}{(\omega-\Omega)^2+\Gamma^2} - \frac{1}{(\omega+\Omega)^2+\Gamma^2}\right] . \tag{5}$$

We now consider the effects of rotating-wave and Markov approximations in computing the correlations in Eq. (2) in the main text from this spectral density.

Intuitively, the rotating wave-approximation neglects terms in which the system decays to a lower state by absorbing energy from the bath (or vice versa) while the Markov approximation (for the interaction between the RC and the residual bath) replaces weak frequency dependencies with their value at resonance. Furthermore, we need to consider that, from the analysis of Eq. (15) in the main text, the residual bath should have both positive and negative frequencies.

In order to impose the rotating-wave approximation[5,6] at positive (negative) frequencies, we neglect the peak at negative (positive) frequencies in the spectral density, i.e., the second (first) term in Supplementary Eq. (5). With this in mind, by inserting Supplementary Eq. (5) into Eq. (2) from the main text, we obtain

$$\begin{aligned} C(t) \simeq\ & \frac{\lambda^2\gamma}{8\pi\Omega}\int_{-\infty}^{\infty} d\omega \frac{\coth[\beta\Omega/2]\cos\omega t - i\sin\omega t}{(\omega-\Omega)^2+\Gamma^2} \\ & - \frac{\lambda^2\gamma}{8\pi\Omega}\int_{-\infty}^{\infty} d\omega \frac{\coth[-\beta\Omega/2]\cos\omega t - i\sin\omega t}{(\omega+\Omega)^2+\Gamma^2} \\ =\ & \frac{\lambda^2}{2\Omega}e^{-\Gamma t}e^{-i\Omega t} \ , \end{aligned} \tag{6}$$

where, in the first step, we both approximated the value of the hyperbolic cotangent at the resonant values $\pm\Omega$, enforcing the Markov approximation[5], and set $\beta \to \infty$. This correlation function is the same as the non-Matsubara part in Eq. (4) in the main text for $\beta \to \infty$.

In summary, first, the interaction in Eq. (16) (in the main text) between the RC mode and the residual bath is forced to obey a rotating-wave approximation (even though such an approximation is not justified). Second, the residual bath spectral density is set as frequency independent such that $J_{\mathrm{flat}}(\omega) = \gamma\Omega$. Applying both approximations, in addition to the standard Born-Markov-secular approximations, leads to the following master equation,

$$\dot{\rho} = -i[H_{\mathrm{RC}}, \rho] + D^{(2)}[\rho] \ , \tag{7}$$

where as before

$$H_{\mathrm{RC}} = \frac{\omega_{\mathrm{q}}}{2}\sigma_z + \frac{\Delta}{2}\sigma_x + \sigma_z \frac{\lambda}{\sqrt{2\omega_0}}(a + a^\dagger) + \omega_0 a^\dagger a \ , \tag{8}$$

and now

$$D^{(2)}[\rho] = \frac{\gamma}{2}[2a\rho a^\dagger - a^\dagger a\rho - \rho a^\dagger a]. \tag{9}$$

Note that in the two master equations, Supplementary Eqs. (1) and (7), the frequency of the RC is ω_0. However, the frequency of the primary oscillating-mode correlation function, and the corresponding pseudomode, is

$$\Omega = [\omega_0^2 - (\gamma/2)^2]^{1/2}. \tag{10}$$

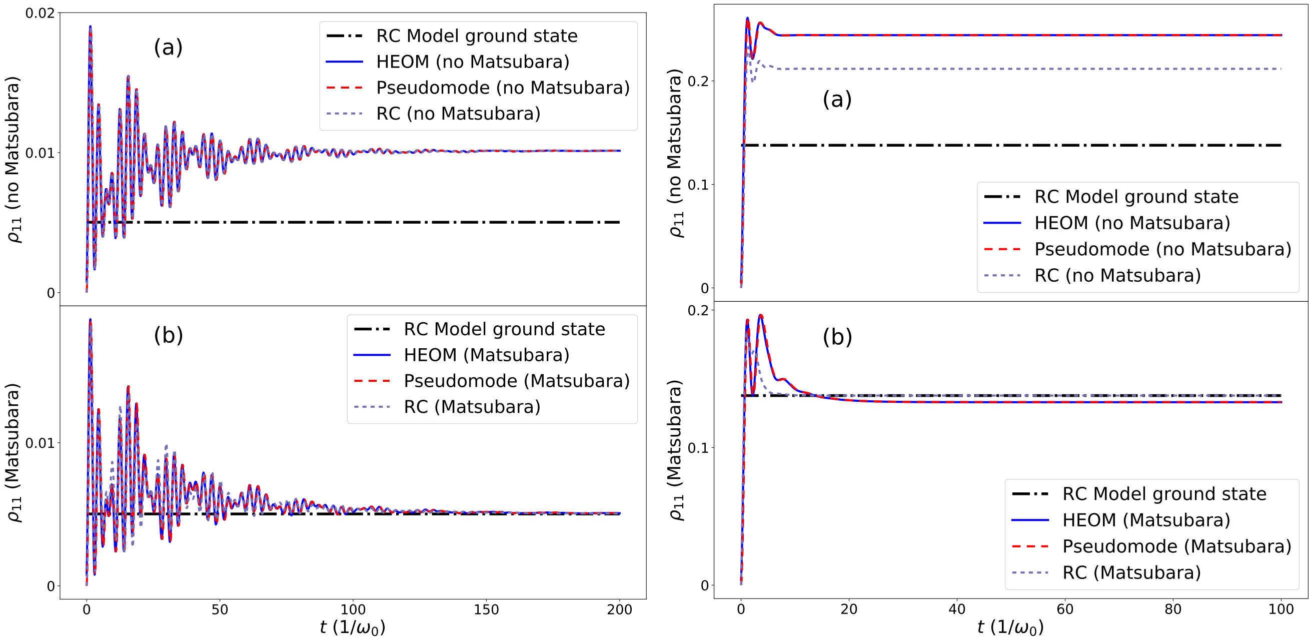

Supplementary Figure 1. **Dynamics of qubit excitation probability.** Probability for the qubit to be in its excited state $\rho_{11} = \langle 1|\rho|1\rangle$, as given by different methods. The left panels use the parameters $\lambda = 0.2\omega_0$, $\gamma = 0.05\omega_0$, $\omega_{\rm q} = 0$, $\Delta = \omega_0$, $T = 0$, as in Fig. 3 in the main text. The right panels use $\lambda = \omega_0$, $\gamma = \omega_0$, as in Fig. 4 in the main text. The curves follow the same labeling scheme as Fig. 3 and Fig. 4.

The difference arises because that renormalized frequency is exact to all orders, while the frequency for the RC mode master equation in contact with the residual bath is only approximate.

Now we can see that the results produced by this "incorrect" derivation of the master equation are, for small γ, exactly the same as the one by the HEOM method where the Matsubara frequencies are ignored, see Supplementary Fig. 1.

Supplementary Note 2: Virtual excitations from auxiliary density operators

Several works[7,8] have explicitly shown how to extract moments of the bath coupling operator $X = \sum_k g_k/\sqrt{2\omega_k}\left(b_k + b_k^\dagger\right)$ and the equivalent sum of mass weighted momenta, $P = i\sum_k g_k\sqrt{\omega_k/2}\left(b_k^\dagger - b_k\right)$ from the ADOs of the HEOM. In the limit of a single (and undamped) mode in the environment, Schinabeck *et al.* [9] showed that the occupation of the (essentially single) bath mode can be extracted from certain second-level ADOs in the hierarchy.

In the general case, we can make progress by making a similar comparison between the HEOM and the equations of motion for the coupling operators for each pseudomode in Eq. (11) in the main text. In the interaction picture, each mode operator rotates as $a_i(t) = a_i \exp(-i\omega_i t)$. The equation of motion for $\mathrm{Tr}_{\rm E}[\lambda_i a_i(t)\rho(t)]$, derived from the Lindbladian master equation given in Eq. (11) and Eq. (12) in the main text, and where $\mathrm{Tr}_{\rm E}[\cdot]$ denotes partial trace over the environment modes, follows as,

$$\begin{aligned}&\frac{d}{dt}\mathrm{Tr}_{\rm E}[\lambda_i a_i(t)\rho(t)] \qquad (11)\\ &= (-i\mathcal{L} - i\Omega - \Gamma)\mathrm{Tr}_{\rm E}[\lambda_i a_i(t)\rho(t)]\\ &- i\left\{\sigma_z \quad \mathrm{Tr}_{\rm E}\left[\lambda_i a_i(t)\left(\sum_k \lambda_k\{a_k(t) + a_k^\dagger(t)\}\right)\rho(t)\right]\right.\\ &\left.- \ \mathrm{Tr}_{\rm E}\left[\left(\sum_k \lambda_k\{a_k(t) + a_k^\dagger(t)\}\right)\lambda_i a_i(t)\rho(t)\right]\sigma_z\right\}.\end{aligned}$$

Here, $\mathcal{L}\rho = -i[H_{\rm s}, \rho]$, where $H_{\rm s}$ is the system part of the Hamiltonian. We can compare this to the equation of motion of $\rho^{0,0,0,1}$ in the HEOM as per Eq. (10) in the main text,

$$\begin{aligned}\frac{d}{dt}\rho^{0,0,0,1} &= (-i\mathcal{L} - i\Omega - \Gamma)\rho^{0,0,0,1} \qquad (12)\\ &- i\left(c_4^R[\sigma_z, \rho] + c_4^I\{\sigma_z, \rho\}\right)\\ &- i\left[\sigma_z \sum_k P_+^k\rho^{0,0,0,1} - P_+^k\rho^{0,0,0,1}\sigma_z\right],\end{aligned}$$

where the operator $P_+^k\rho^n = \rho^{n_k+1}$ raises the kth element of n by one. Similar equations can be derived for the other first-tier ADOs and we can immediately make a correspondence between the two equations, such that $\left\langle a_1^\dagger a_1\right\rangle = \rho_{0,0,1,1}/\lambda_1^2$. Note that the non-Matsubara

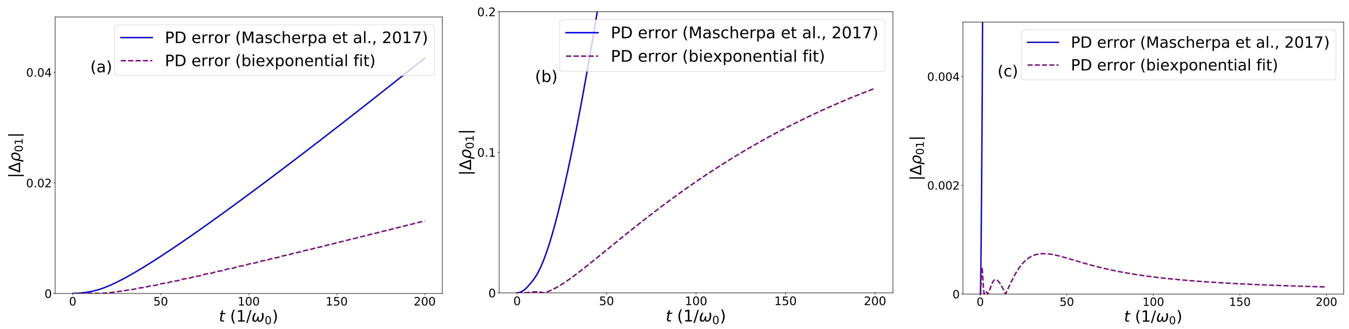

Supplementary Figure 2. **Error analysis using the pure dephasing solution**. Error in the dynamics given by the coherence ρ_{01} term of the density matrix in the σ_z basis by considering a pure dephasing model. We compare the error according to Ref. [10] against the error due to our Matsubara fitting approach. The error due to the fitting is computed by simulating the dynamics exactly by taking the full infinite Matsubara integral and then by considering only two terms from the fitting and finding the difference in the dynamics. In the left figure (a), $\lambda = 0.2\omega_0$, $\gamma = 0.05\omega_0$, $\omega_q = 0$, $\Delta = 0$, $T = 0$. In the center figure (b) $\lambda = 0.4\omega_0$, $\gamma = 0.4\omega_0$. In the right figure (c) $\lambda = \omega_0$, $\gamma = \omega_0$. We see that in all cases, at long times, the dynamics is very sensitive to the error. In the very broad-bath case (c) the performance in comparing to the pure dephasing results is misleading since the suppression of the error is just due to the very fast decay of the coherences.

pseudomode is associated with the last two indices, corresponding to $a_1(t)$ and $a_1^\dagger(t)$, while the two Matsubara modes, being zero frequency modes, are just associated with a single index each.

Supplementary Note 3: Error bounds from fitting

The error due to the numerical fitting of the infinite Matsubara sum with the biexponential in Eq. (9) of the main text will inevitably lead to an error in the dynamics of the system. This has been discussed extensively in Mascherpa *et al.*[10] where it was argued that an error in the correlation function, $\Delta C(t)$, leads to a corresponding error in the expectation of any operator which is bound by the inequality,

$$|\,\Delta\langle\hat{\mathcal{O}}(t)\rangle\,| \leq ||\,\hat{\mathcal{O}}\,||\,\left(e^{\int_0^t dt' \int_0^{t'} dt'' |\Delta C(t'-t'')|} - 1\right) \quad (13)$$

where $||\,\hat{\mathcal{O}}\,||$ denotes the operator norm. In this section we consider whether this result is useful to characterize the error in the dynamics of our model in the main text. Before showing that result, we first discuss another comparison we will make: the exactly solvable pure-dephasing model.

Pure dephasing model

The pure dephasing case is given by the condition $\Delta = 0$ in the Hamiltonian in Eq. (1) of the main text. Since the pure dephasing case has an analytical solution, we can in principle also use it as a benchmark for comparing errors. The evolution of the density matrix is given, in the σ_z basis, by[11],

$$\rho = \begin{bmatrix} \rho_{00}(0) & \rho_{01}(0)e^{-F(t)} \\ \rho_{10}(0)e^{-\bar{F}(t)} & \rho_{11}(0) \end{bmatrix} ,$$

with $F(t) = i\omega_q t + \int_0^t d\tau D(\tau)$, and $D(\tau)$ is defined as,

$$D(\tau) = 2\int_0^\tau ds\left[C(\tau - s) + \bar{C}(\tau - s)\right] , \quad (14)$$

where $C(t)$ is the correlation function.

Let us write $C(t) = \sum_k c_k \exp(\mu_k t)$, where c_k and μ_k can be real or imaginary (note here that c_k and μ_k refer to a generic decomposition of the correlation functions, not the one we define in the main text). This allows us now to write $D(\tau)$ as a sum of exponentials as well. After integrating, we again obtain a sum of exponentials

$$\int_0^\tau dsC(\tau - s) = \int_0^\tau ds \sum_k c_k e^{\mu_k(\tau-s)}$$
$$= \sum_k c_k \left[\frac{e^{\mu_k\tau} - 1}{\mu_k}\right] . \quad (15)$$

Using this expression in Supplementary Eq. (14), we can write

$$D(\tau) = 2\sum_k \frac{c_k}{\mu_k}(e^{\mu_k\tau} - 1) + \text{H.c.} , \quad (16)$$

which gives

$$\int_0^t d\tau D(\tau) = 2\sum_k \left[\frac{c_k}{\mu_k^2}(e^{\mu_k t} - 1) - \frac{c_k}{\mu_k}t\right] + \text{H.c.} . \quad (17)$$

Now, for any correlation function which is a sum of exponentials we can easily write down the evolution as two

parts: the sum of exponents from the non-Matsubara part and an integral taking into account the full Matsubara contribution, $\int_0^t D(\tau) = \int_0^t D_0(\tau) + \int_0^t D_{\rm m}(\tau)$. In our case, the Matsubara terms are already given as an infinite sum of exponentials and in the zero temperature limit this can be written as,

$$\begin{aligned}\int_0^t dt D_{\rm m}(\tau) &= 4\int_0^t d\tau \int_0^\tau ds \left[-\frac{4\lambda^2\gamma}{\pi}\left(\frac{\pi}{\beta}\right)^2 \sum_{n=1}^{\infty} \frac{n\exp(-2n\pi s/\beta)}{[(\Omega+i\Gamma)^2+(2n\pi/\beta)^2][(\Omega-i\Gamma)^2+(2n\pi/\beta)^2]}\right] \\ &= -\frac{4\lambda^2\gamma}{\pi}\int_0^t d\tau \int_0^\tau ds \int_0^\infty dx \, \frac{x\exp(-xs)}{[(\Omega+i\Gamma)^2+x^2][(\Omega-i\Gamma)^2+x^2]} \\ &= -\frac{4\lambda^2\gamma}{\pi}\int_0^\infty dx \, \frac{1}{[(\Omega+i\Gamma)^2+x^2][(\Omega-i\Gamma)^2+x^2]}\left(t+\frac{\exp(-xt)-1}{x}\right) \quad ,\end{aligned}$$

where we took $2n\pi/\beta \to x$. We use this expression to compare the dynamics of the pure dephasing model for the full Matsubara contribution against our approximation using just two exponents. In Supplementary Fig. 2 we show the comparison for different parameter regimes, and the bound in the same system quantities given by the inequality in Supplementary Eq. (13).

Unfortunately, it becomes apparent from the figure that both the bound proposed in Ref. [10], and the pure dephasing result, are exponentially sensitive to errors in the fit at long times (when the error is comparable to the evolution time), which in the main text is one of the regimes we are interested in. However, it turns out that in terms of the influence of an error in the correlation functions on the system dynamics, the pure dephasing case is the worst case, as discussed in Mascherpa *et al.*[10], and hence, unfortunately, these results do not give us much information about the potential error in results away from the regime $\Delta = 0$. A potential alternative method to characterize stability and error of results is discussed in the next section.

Supplementary Note 4: Sensitivity of the dynamics to perturbations

In order to further evaluate the sensitivity of the dynamics and steady-state to the quality of the fitting of the Matsubara terms for $\Delta \neq 0$, we numerically compute the evolution with small random perturbations added to the fit parameters. We use the standardized measure of dispersion of a distribution, the coefficient of variation, to quantify how much the steady-state population varies as we inject random perturbations to the parameters of our fitting.

The coefficient of variation is defined as the ratio between the standard deviation and mean (σ/μ) of observations. In this case, the observations are the steady-state populations of the system density matrix. The parameters that we will perturb are the amplitudes of the biexponentials (c_1, c_2) and the frequencies (μ_1, μ_2) in Eq. (9) of the main text. We inject perturbations as follows,

$$\begin{aligned} c_i &\to c_i(1+\delta) \\ \mu_i &\to \mu_i(1+\delta), \end{aligned}$$

where $\delta \in [-\delta_{\rm max}, \delta_{\rm max}]$ is the perturbation in the parameters with maximum absolute value $\delta_{\rm max}$. In Supplementary Fig. 3, we plot (σ/μ) against randomly picked values of δ from a uniform distribution and then compute the statistics after 200 runs.

The intuition here is that these results show that additional small perturbations (errors) in the fitting parameters do not give a large variance in the results. Given that we also know the error in the fit without these additional perturbations, these results give us an intuition about how that error influences the steady-state of the system (see the next section for an example). Primarily however, these results show that as the perturbations/errors are decreased, the coefficient of variation for the steady state populations also decreases, suggesting that we can place a qualitative error bound on the final results.

Supplementary Note 5: Steady-state as a function of coupling strength

As discussed in the main text, as the coupling strength increases, the HEOM and pseudomode predictions diverge from that of the RC model. We also note that, as the coupling increases, the Matsubara terms become more important. To clarify this, and give an example for the error analysis performed in the previous section, we compare the steady-state system excitation probability and the bath-mode photon population as a function of the coupling strength at zero temperature, see Supplementary Fig. 4.

We will try to make a qualitative argument here regarding the difference in the RC, HEOM/pseudomode predictions. Our sensitivity analysis in the previous section suggests that potential perturbations, or errors, in the fitting of the Matsubara terms can lead to errors

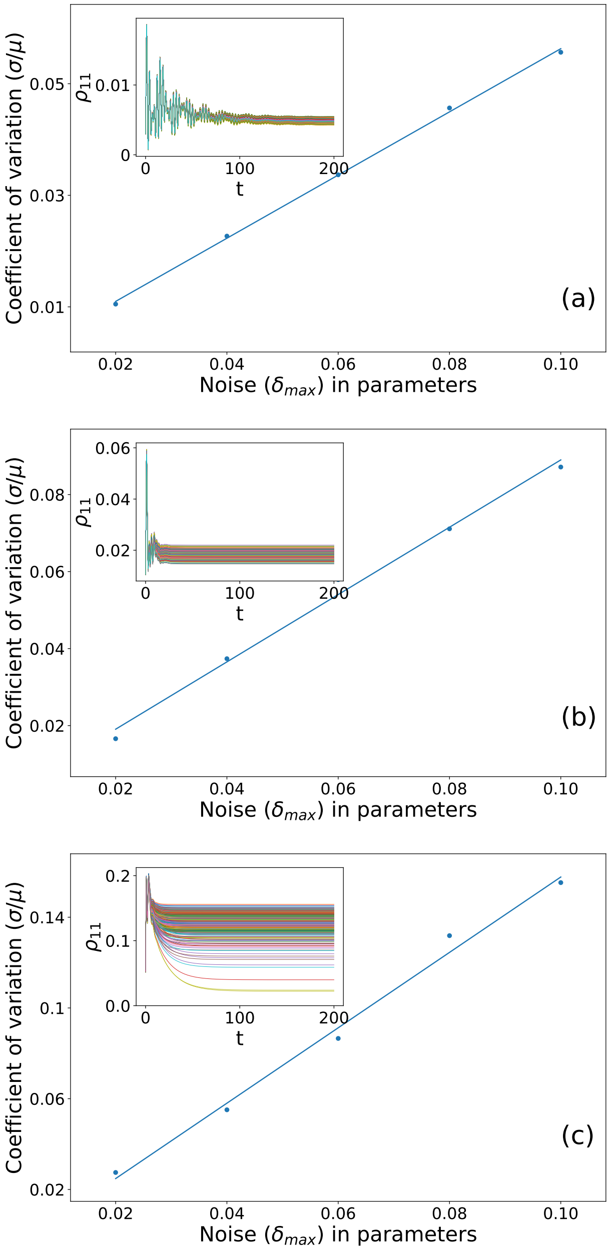

Supplementary Figure 3. **Error analysis based on uncertainties in the fit**. Here we show the coefficient of variation, σ/μ, of the steady-state excited state population of the qubit against injected perturbations in the parameters of the biexponential fitting ($\pm\delta_{\rm max}$). In the top panel (a) $\lambda = 0.2\omega_0$, $\gamma = 0.05\omega_0$, $\omega_{\rm q} = 0$, $\Delta = \omega_0$, and $T = 0$. In the center panel (b) $\lambda = 0.4\omega_0$ and $\gamma = 0.4\omega_0$. In the bottom panel (c) $\lambda = \omega_0$ and $\gamma = \omega_0$. These results are averaged over 200 random choices of perturbed parameters. In the insets we show examples of the dynamics for perturbations up to 10% in the parameters. As the perturbations decrease, we obtain less deviation in the steady-state populations. Note that in the inset in figure (c) we have removed examples which generate unphysical system behavior, which can occur for large perturbations in the fitting parameters.

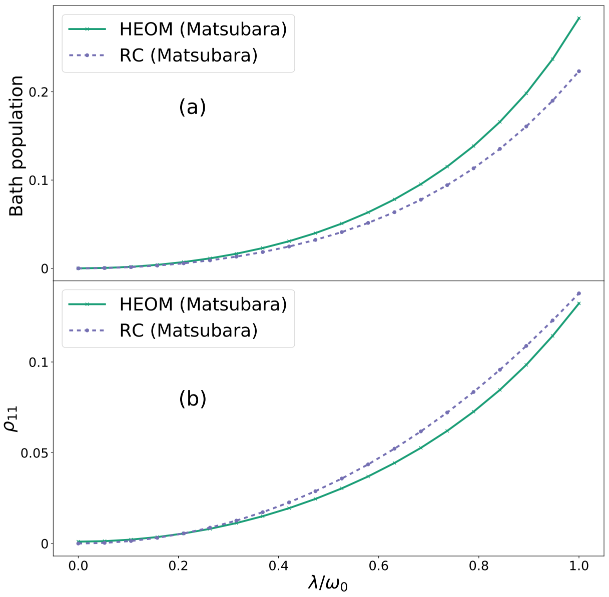

Supplementary Figure 4. **Steady-state properties as a function of the coupling strength**. In the upper figure we plot the steady state population of the relevant effective "bath mode" against the coupling strength λ, for $\omega_{\rm q} = 0$, $\Delta = \omega_0$, $T = 0$, and $\gamma = \omega_0$. In the lower figure we plot the qubit excited state probability for the same parameters.

in the steady-state population. From a direct comparison between the fit we use in this data, we estimate the parameter error in the fit to be about 1%. As we see from Supplementary Fig. 3 an additional injected error of 1–2% introduces a variance in the results at most 2–4%, even in the USC regime. However, in Supplementary Fig. 4 we see that the difference in the RC versus HEOM/pseudomode results are much larger than this potential error from the inaccuracy in the fit (especially in the broad-bath case, see Supplementary Fig. 1).

Thus, it would be reasonable to believe that this difference is not just an artifact of a poor fitting of the Matsubara terms but comes more from the RC approach being fundamentally inadequate in capturing the full correlations between the qubit and its environment for broad baths and strong couplings. In addition, this reasoning suggests the fitting procedure we employ here can give reliable predictions up to a potential error of 2–4% in the populations in the long-time limit for the most difficult parameter choices (broad baths and strong couplings).

One other interesting error-related point in Supplementary Fig. 4 is the fact that the system population does not go perfectly to zero as $\lambda \rightarrow 0$ (the smallest value of λ actually used in this figure is $1 \times 10^{-5}\omega_0$). This is because, as we saw in Fig. 2 of the main text, at weak couplings we still need the Matsubara terms to give a correct detailed balance. An equivalent plot without the Matsubara terms results in a residual excited state population of $\rho_{11} \approx 0.055$, for

$\lambda = 1 \times 10^{-5}\omega_0$, whereas with the Matsubara terms included, that population extracted from the HEOM solution is 0.001. In principle this small residual "effective temperature" is another indication of the quality of the fit, at least for small coupling strengths.

Supplementary Note 6: Generalized pseudomodes

In a seminal work[12], Garraway introduced the idea of modelling the dynamics of an open quantum system by replacing the environment with a set of bosonic pseudomodes. This can simplify the original problem in two ways. First, the infinite environmental degrees of freedom in the original system can be replaced by a finite set of modes. Second, the time-evolution of the pseudomodes can be captured by a Lindblad master equation. However, in his examples, Garraway restricted himself to a rotating-wave-approximation form for the interaction between system and environment, and single excitations. Recently, his proof was formally extended by Tamascelli *et al.*[13,14] to allow for non-RWA interactions. However, here we need to adapt their proof to deal with the problem we face in our main text; what happens if the correlation functions are negative?

In this section, we adapt the results in Ref. [13] to explicitly write a pseudomodes-model valid when the correlations of the original (Gaussian) bath can be written as a weighted-sum of exponentials. We show that when some of these weights are negative, the exact system dynamics corresponds to a pseudomode model involving a modified quantum-mechanical equation of motion with a non-Hermitian Hamiltonian. Since approximating the Matsubara correlations in our main text with exponentials requires negative weights, this result has particular relevance in terms of restoring the correct non-Markovian and equilibrium physics.

After modelling the correlation function of the original spin-boson model as a sum of N exponentials, we proceed in three steps. First, we map the system dynamics to the situation in which the spin interacts with N independent harmonic baths. Importantly, these baths follow a non-standard equation of motion when their Hamiltonian is non-Hermitian. Second, we show that each of these baths can be replaced by a non-Hermitian open quantum system involving a single pseudomode. The spectral density characterizing the interaction between each pseudomode and its residual environment is found to be constant for all positive and negative frequencies. Third, we show that this open quantum system is equivalent to imposing a pseudo-Schrödinger master equation for each pseudomode.

We stress that the steps above extend the work done in Ref. [13] and we restrict ourselves to zero-temperature.

From one bath to N baths

To set the notation, as in the main text we consider a system S interacting with an environment B of bosonic modes under the Hamiltonian

$$H = H_S + H_B + \sigma_z \tilde{X} \ , \tag{18}$$

where the interaction operator is $\tilde{X} = \sum_k \tilde{X}_k$, $\tilde{X}_k = g_k/\sqrt{2\omega_k}(b_k + b_k^\dagger)$, and b_k is the annihilation operators of the kth bath mode with energy ω_k. The Hamiltonian of the system and bath can be chosen to be $H_S = (\omega_\mathrm{q}/2)\ \sigma_z + (\Delta/2)\ \sigma_x$ and $H_B = \sum_k \omega_k b_k^\dagger b_k$, respectively, as in Eq. (1) in the main text. Importantly, we assume the initial state to be factorized as $\rho_S(0) \otimes \rho_B(0)$, where $\rho_S(0)$ is the initial state of the system, and where $\rho_B(0)$ is a Gaussian state of the bath satisfying $\mathrm{Tr}_B[\tilde{X}\rho_B(0)] = 0$. The reduced evolution of the system $\rho_S(t) = \mathrm{Tr}_B[\rho(t)]$ can be written as

$$\begin{aligned}\rho_S(t) \;=\; & \sum_{n=0}^{\infty}(-i)^n \int_0^t dt_1 \cdots \int_0^{t_{n-1}} dt_n \sum_{n'=0}^{\infty}(i)^{n'} \int_0^t dt_1' \cdots \int_0^{t'_{n'-1}} dt'_{n'} \\ & \mathrm{Tr}_B\left(\tilde{X}(t_1)\cdots\tilde{X}(t_n)\rho_B(0)\tilde{X}(t'_{n'})\cdots\tilde{X}(t'_1)\right) U_0(t)\sigma_z(t_1)\cdots\sigma_z(t_n)\rho_S(0)\sigma_z(t'_{n'})\cdots\sigma_z(t'_1)U_0^\dagger(t) \ ,\end{aligned} \tag{19}$$

where $\tilde{X}(t) = \exp(iH_Bt)\tilde{X}\exp(-iH_Bt)$, and $\sigma_z(t) = U_0^\dagger(t)\sigma_z U_0(t)$, with $U_0(t) = \exp(-iH_St)$. Since the initial state of the bath is Gaussian and such that $\mathrm{Tr}_B[\tilde{X}\rho_B(0)] = 0$, the correlations $\mathrm{Tr}_B\left(\tilde{X}(t_1)\cdots\tilde{X}(t_n)\rho_B(0)\tilde{X}(t'_{n'})\cdots\tilde{X}(t'_1)\right)$ appearing in the equation above can, in principle, be retrieved from the two-time correlation

$$C(t) = \mathrm{Tr}_B[\tilde{X}(t)\tilde{X}(0)] \ . \tag{20}$$

For this reason, the reduced Dyson equations in Supplementary Eq. (19) is invariant under splitting of the original bath B into N independent copies B_i [with initial Gaussian state $\rho_{B_i}(0)$] described by the total Hamiltonian

$$H' = H_S + \sum_{i=1}^{N} H'_{B_i} + \sigma_z \sum_{i=1}^{N} \tilde{X}_i \ , \tag{21}$$

and such that the two-time correlation functions are constrained by

$$\mathrm{Tr}_{B_1}\cdots\mathrm{Tr}_{B_N}\left[\left(\prod_{i=1}^{N}\rho_{B_i}(0)\right)\sum_{i=1}^{N}\tilde{X}_i(t)\sum_{j=1}^{N}\tilde{X}_j(0)\right]=C(t), \tag{22}$$

where $C(t)$ is the original correlation function in Supplementary Eq. (20). In the equations above, H'_{B_i} and $\tilde{X}_i$ are the free-bath Hamiltonian and coupling operator with support on the bath B_i. Note that, as before, the time dependence in Supplementary Eq. (22) follows the free-bath Hamiltonian $\tilde{X}_i(t) = \exp(iH'_{B_i})\tilde{X}_i\exp(-iH'_{B_i}t)$. Since the baths are independent, the constraint in Supplementary Eq. (22) can be written as

$$\begin{aligned} C(t) \; = \; & \sum_{i=1}^{N}\mathrm{Tr}[\rho_{B_i}(0)\tilde{X}_i(t)\tilde{X}_i(0)] \\ & +\sum_{i\neq j}\mathrm{Tr}_{B_i}\left[\rho_{B_i}(0)\tilde{X}_i(t)\right]\mathrm{Tr}_{B_j}\left[\rho_{B_i}(0)\tilde{X}_j(0)\right]. \end{aligned} \tag{23}$$

To satisfy the equation above it is sufficient to impose

$$\begin{aligned} \sum_{i=1}^{N}\mathrm{Tr}_{B_i}[\rho_{B_i}(0)\tilde{X}_i(t)\tilde{X}_i(0)] &= C(t) \\ \mathrm{Tr}_{B_i}\left(\rho_{B_i}(0)\tilde{X}_i(0)\right) &= 0 \qquad \forall i=1,\cdots,N \ . \end{aligned} \tag{24}$$

The simplicity of decomposing the original bath into N independent ones as just described hides an important point. In fact, since we are only interested in the dynamics of the reduced system $\rho_S(t)$, we can let the coupling operators $\tilde{X}_i$ (and hence H') to be non-Hermitian, as long as they satisfy the contraints in Supplementary Eq. (24) and give rise to equations of motion in the same form as in Supplementary Eq. (19) with the substitution $\tilde{X}\mapsto\sum_{i=1}^{N}\tilde{X}_i$. To ensure the latter, we need to impose the equation of motion

$$\frac{d}{dt}\rho'(t) = -i[H',\rho'(t)] \ . \tag{25}$$

We here explicitly stress that, for a non-Hermitian Hamiltonian H' the usual Shrödinger dynamics would imply the right-hand side of the previous equation to take the form $-i[H'\rho'(t)-\rho'(t)H'^{\dagger}]$. Here, however, in order to ensure the invariance of the Dyson equation, we need to impose Supplementary Eq. (25) instead. Under these hypothesis

$$\rho'_S(t)=\rho_S(t) \ , \tag{26}$$

where $\rho'_S = \mathrm{Tr}_{B_1}\cdots\mathrm{Tr}_{B_N}[\rho'(t)]$.

From N baths to N pseudomodes

Following Ref. [13], we now can proceed a step further to show that each of the baths B_i can be replaced by a single pseudomode (associated to a Hilbert space R_i, annihilation operator a_i, and frequency Ω_i) interacting with a residual environment E_i (whose modes are associated with annihilation operators $b_{i,\alpha}$ and have frequency $\omega_{i,\alpha}$) so that the full Hamiltonian now reads

$$H'' \; = \; H_S + H''_B + \sigma_z\sum_{i=1}^{N}\tilde{X}_i^a \ , \tag{27}$$

where $\tilde{X}_i^a = \lambda_i/\sqrt{2\Omega_i}(a_i^\dagger + a_i)$, with the parameters λ_i setting the scale for the interaction between the pseudomodes and the system. We also defined the free-bath Hamiltonian as

$$H''_B = \sum_{i=1}^{N}H''_{B_i} \ , \tag{28}$$

where

$$\begin{aligned} H''_{B_i} \; = \; & \Omega_i a_i^\dagger a_i + i\sum_{\alpha}\frac{g_{i,\alpha}}{\sqrt{2\omega_{i,\alpha}}}(b_{i,\alpha}^\dagger a_i - a_i^\dagger b_{i,\alpha}) \\ & +\sum_{\alpha}\omega_{i,\alpha}b_{i,\alpha}^\dagger b_{i,\alpha} \ . \end{aligned} \tag{29}$$

The interaction of each pseudomode with its residual environment E_i is described by the parameters $g_{i,\alpha}$ which, in the continuum limit, are characterized by the spectral densities

$$J_i(\omega) \; = \; \pi\sum_{\alpha}\frac{g_{i,\alpha}^2}{2\omega_{i,\alpha}}\delta(\omega-\omega_{i,\alpha}) \ . \tag{30}$$

We now impose the pseudo-equation of motion [see Supplementary Eq. (25)]

$$\frac{d}{dt}\rho''(t) = -i[H'',\rho''(t)] \ , \tag{31}$$

where we stress again that, since the Hamiltonian H'' can, in principle, be non-Hermitian, these equations might be non-standard. Analogously to Supplementary Eq. (24), we also impose the following constraints on the correlations

$$\begin{aligned} \sum_i \mathrm{Tr}_{R_i}\mathrm{Tr}_{E_i}[\rho_{R_i}(0)\rho_{E_i}(0)\tilde{X}_i^a(t)\tilde{X}_i^a(0)] &= C(t) \\ \mathrm{Tr}_{R_i}\mathrm{Tr}_{E_i}\left(\rho_{R_i}(0)\rho_{E_i}(0)\tilde{X}_i^a(0)\right) &= 0 \ , \end{aligned} \tag{32}$$

for an initial environmental state of the form $\prod_i[\rho_{R_i}(0)\rho_{E_i}(0)]$, where $\rho_{R_i}(0)$ and $\rho_{E_i}(0)$ are the initial Gaussian state of the ith pseudomode and its residual environment, respectively. Importantly, we assume these states to be invariant under the time evolution induced by the free Hamiltonian of the bath

H''_B. In the expression above, the time evolution follows $\tilde{X}^a_i(t) = \exp(iH''_{B_i}t)\tilde{X}^a_i \exp(-iH''_{B_i}t)$. Following the same considerations as above, on the equivalence between two open quantum systems, Supplementary Eq. (31) and Supplementary Eq. (32) are sufficient to induce a Dyson equation equivalent to the original one in Supplementary Eq. (19), provided the process is Gaussian. In turn, the equivalence of the Dyson equations also implies the reduced dynamics $\rho''_S(t) = \mathrm{Tr}_R\mathrm{Tr}_E[\rho''(t)]$ (where $R = \prod_i R_i$ and $E = \prod_i E_i$) to exactly match the original one, i.e.,

$$\rho''_S(t) = \rho'_S(t) = \rho_S(t) \quad . \tag{33}$$

Note that this result depends on the free Hamiltonian in Supplementary Eq. (29) only implicitly through the definition [which follows the pseudo-Schrödinger equation in Supplementary Eq. (31)] of the interaction picture required in Supplementary Eq. (32). This could allow, in principle, to consider different free dynamics to prove the same equivalence as long as the whole process is Gaussian.

From Supplementary Eq. (32), we see that the correlation $C(t)$ effectively induces constraints on the spectral densities in Supplementary Eq. (30) and the couplings λ_i. Specifically, choosing $J_i(\omega)$ to be constant for both positive and negative frequencies, i.e.,

$$J_i(\omega) \;=\; \frac{\gamma_i}{2} \quad , \tag{34}$$

and assuming all the pseudomodes and their residual environments to be initially in their vacuum state, the reduced dynamics of the system is the same as that of the original spin-boson model with correlations

$$C(t) = \sum_{i=1}^{N} \frac{\lambda_i^2}{2\Omega_i} \exp\left[-(i\Omega_i + \gamma_i/2)t\right] \quad . \tag{35}$$

To show this, we solve the Heisenberg equation of motion for the free bath and insert the result in Supplementary Eq. (32). We start by noticing that the equal-time commutation relations $[b_{i,\alpha}(t), b^\dagger_{j,\beta}(t)] = \delta_{ij}\delta_{\alpha\beta}$, $[a_i(t), a^\dagger_j(t)] = \delta_{ij}$, and $[b_{i,\alpha}(t), a_i(t)] = [b_{i,\alpha}(t), a^\dagger_i(t)] = 0$ are satisfied once we impose them as an initial condition (the dynamics of the open quantum system is unitary). We can now formally write the equations of motion for the residual environments E_i as

$$\frac{d}{dt} b_{i,\alpha} = i[H''_B, b_{i,\alpha}] = -i\omega_{i,\alpha} b_{i,\alpha} + \frac{g_{i,\alpha}}{2\sqrt{\omega_{i,\alpha}}} a_i \quad , \tag{36}$$

which leads to the following equations for the corresponding Laplace transforms (denoted by an overhead bar)

$$\begin{aligned}
\bar{b}^\dagger_{i,\alpha} + \bar{b}_{i,\alpha} \;&=\; \left(\frac{b^\dagger_{i,\alpha}(0)}{s - i\omega_{i,\alpha}} + \frac{b_{i,\alpha}(0)}{s + i\omega_{i,\alpha}}\right) \\
&\quad + \frac{g_{i,\alpha}\left[s(\bar{a}^\dagger_i + \bar{a}_i) + i\omega_{i,\alpha}(\bar{a}^\dagger_i - \bar{a}_i)\right]}{\sqrt{2\omega_{i,\alpha}}(s^2 + \omega^2_{i,\alpha})} \\
\bar{b}^\dagger_{i,\alpha} - \bar{b}_{i,\alpha} \;&=\; \left(\frac{b^\dagger_{i,\alpha}(0)}{s - i\omega_{i,\alpha}} - \frac{b_{i,\alpha}(0)}{s + i\omega_{i,\alpha}}\right) \\
&\quad + \frac{g_{i,\alpha}\left[s(\bar{a}^\dagger_i - \bar{a}_i) + i\omega_{i,\alpha}(\bar{a}^\dagger_i + \bar{a}_i)\right]}{\sqrt{2\omega_{i,\alpha}}(s^2 + \omega^2_{i,\alpha})} \quad ,
\end{aligned} \tag{37}$$

where s is the complex variable introduced by the Laplace transformation. Similarly, we can write the Heisenberg equations for the pseudomodes as

$$\dot{a}_i = i[H''_B, a_i] = -i\Omega_i a_i - \sum_\alpha \frac{g_{i,\alpha}}{\sqrt{2\omega_{i,\alpha}}} b_{i,\alpha} \quad , \tag{38}$$

which, after a Laplace transform, become

$$\begin{aligned}
s\bar{x}_i \;&=\; x_i(0) + \Omega_i \bar{p}_i - \sum_\alpha \frac{g_{i,\alpha}}{\sqrt{2\omega_{i,\alpha}}}(\bar{b}^\dagger_{i,\alpha} + \bar{b}_{i,\alpha}) \\
s\bar{p}_i \;&=\; p_i(0) - \Omega_i \bar{x}_i - i\sum_\alpha \frac{g_{i,\alpha}}{\sqrt{2\omega_{i,\alpha}}}(\bar{b}^\dagger_{i,\alpha} - \bar{b}_{i,\alpha}) \quad ,
\end{aligned} \tag{39}$$

in terms of the dimensionless quadratures $x_i = a^\dagger_i + a_i$ and $p_i = i(a^\dagger_i - a_i)$. By inserting Supplementary Eq. (37) into Supplementary Eq. (39) we finally obtain

$$\begin{aligned}
s\bar{x}_i \;&=\; x_i(0) + \left(\Omega_i - \sum_\alpha \frac{g^2_{i,\alpha}}{2(s^2 + \omega^2_{i,\alpha})}\right)\bar{p}_i \\
&\quad - s\sum_\alpha \frac{g^2_{i,\alpha}}{2\omega_{i,\alpha}(s^2 + \omega^2_{i,\alpha})}\bar{x}_i - x^{\mathrm{in}}_i \\
s\bar{p}_i \;&=\; p_i(0) - \left(\Omega_i - \sum_\alpha \frac{g^2_{i,\alpha}}{2(s^2 + \omega^2_{i,\alpha})}\right)\bar{x}_i \\
&\quad - s\sum_\alpha \frac{g^2_{i,\alpha}}{2\omega_{i,\alpha}(s^2 + \omega^2_{i,\alpha})}\bar{p}_i - p^{\mathrm{in}}_i \quad ,
\end{aligned} \tag{40}$$

where

$$\begin{aligned}
x^{\mathrm{in}}_i \;&=\; \sum_\alpha \frac{g_{i,\alpha}}{\sqrt{2\omega_{i,\alpha}}}\left(\frac{b^\dagger_{i,\alpha}(0)}{s - i\omega_{i,\alpha}} + \frac{b_{i,\alpha}(0)}{s + i\omega_{i,\alpha}}\right) \\
p^{\mathrm{in}}_i \;&=\; i\sum_\alpha \frac{g_{i,\alpha}}{\sqrt{2\omega_{i,\alpha}}}\left(\frac{b^\dagger_{i,\alpha}(0)}{s - i\omega_{i,\alpha}} - \frac{b_{i,\alpha}(0)}{s + i\omega_{i,\alpha}}\right) \quad .
\end{aligned} \tag{41}$$

Using Supplementary Eq. (30), we can write Supplementary Eq. (40) in the continuum limit as

$$\begin{aligned}
s\bar{x}_i \;&=\; x_i(0) + \left[\Omega_i - \int_{-\infty}^{\infty} d\omega \frac{J_i(\omega)\omega}{\pi(s^2 + \omega^2)}\right]\bar{p}_i \\
&\quad - s\int_{-\infty}^{\infty} \frac{J_i(\omega)}{\pi(s^2 + \omega^2)}\bar{x}_i - x^{\mathrm{in}}_i \\
s\bar{p}_i \;&=\; p_i(0) - \left[\Omega_i - \int_{-\infty}^{\infty} d\omega \frac{J_i(\omega)\omega}{\pi(s^2 + \omega^2)}\right]\bar{x}_i \\
&\quad - s\int_{-\infty}^{\infty} \frac{J_i(\omega)}{\pi(s^2 + \omega^2)}\bar{p}_i - p^{\mathrm{in}}_i \quad .
\end{aligned} \tag{42}$$

By inserting Supplementary Eq. (34) into the equation above we obtain

$$\begin{aligned} s\bar{x}_i &= x_i(0) + \Omega_i \bar{p}_i - \gamma_i/2\ \bar{x}_i - x_i^{\text{in}} \\ s\bar{p}_i &= p_i(0) - \Omega_i \bar{x}_i - \gamma_i/2\ \bar{p}_i - p_i^{\text{in}} \ . \end{aligned} \tag{43}$$

Note that Supplementary Eq. (28) seems not to have the correct renormalization terms for the frequency of the pseudomodes. However, this is justified by the choice of spectral densities in Supplementary Eq. (34) as the additional term which renormalizes the frequencies in Supplementary Eq. (42) is $\lim_{\Lambda\to\infty}\int_{-\Lambda}^{\Lambda} d\omega \frac{J_i(\omega)\omega}{\pi(s^2+\omega^2)} = 0$, where we regularized the integral at infinity. For this reason, the frequencies Ω_i already correspond to the correctly renormalized ones. Using the equation above, we find the following equation for the Laplace transform of the quadratures x_i

$$[(s+\gamma_i/2)^2+\Omega_i^2]\bar{x}_i = (s+\gamma_i/2)[x_i(0)-x_i^{\text{in}}]+\Omega_i[p_i(0)-p_i^{\text{in}}], \tag{44}$$

which we can insert into the first of Supplementary Eq. (32) to finally obtain the correlation function as

$$\begin{aligned} C(t) &= \sum_{i=1}^N \frac{\lambda_i^2}{2\Omega_i}\mathcal{L}_t^{-1}\left\{\text{Tr}_{R_i}\text{Tr}_{E_i}[\rho_{R_i}(0)\rho_{E_i}(0)\bar{x}_i x_i(0)]\right\} \\ &= \sum_{i=1}^N \frac{\lambda_i^2}{2\Omega_i}\frac{1}{2\pi i}\int ds \left\{ \frac{[s+\gamma_i/2]\langle x(0)x(0)\rangle_{R_i}}{(s+\gamma_i/2)^2+\Omega_i^2}e^{st} \right. \\ &\quad \left. + \frac{\Omega_i\langle p(0)x(0)\rangle_{R_i}}{(s+\gamma_i/2)^2+\Omega_i^2}e^{st}\right\} \\ &= \sum_{i=1}^N \frac{\lambda_i^2}{2\Omega_i}\exp\left\{-(i\Omega_i+\gamma_i/2)t\right\} \ , \end{aligned} \tag{45}$$

where $\tilde{X}_i^a = \lambda_i/\sqrt{2\Omega_i}x_i$ and we defined $\langle\cdot\rangle_{R_i} \equiv \text{Tr}_{R_i}[\ \cdot\ \rho_{R_i}(0)]$ as the trace over the ith pseudomode, and $\mathcal{L}_t^{-1}$ as the inverse Laplace transform. We further assumed $\rho_{R_i}(0)$ to be the pseudomodes' vacuum state so that $\langle x_i(0)\rangle_{R_i} = 0$, together with $\langle x_i(0)x_i(0)\rangle_{R_i} = 1$ and $\langle p_i(0)x_i(0)\rangle_{R_i} = -i$. This correlation is the same as the one in Supplementary Eq. (35) which is the result we wanted to prove to deduce Supplementary Eq. (33).

The pseudo-Schrödinger equation

Following Refs. [13] and [15], we can now complete the third step promised at the beginning of this section, i.e., showing that the reduced dynamics of the system can be obtained by considering the following effective equation of motion for the system and the pseudomodes R_i

$$\frac{d}{dt}\rho_{\text{pm}} = L[\rho_{\text{pm}}] \ , \tag{46}$$

where $L[\rho] = -i[H_{\text{pm}},\rho] + \sum_i D_i[\rho]$, where the pseudomodes Hamiltonian reads

$$H_{\text{pm}} = H_S + \sigma_z \sum_i \tilde{X}_i^a + \sum_{i=1}^N \Omega_i a_i^\dagger a_i \ , \tag{47}$$

and where $D_i[\rho] = \gamma_i/2\left[2a_i\rho a_i^\dagger - (a_i^\dagger a_i\rho + \rho a_i^\dagger a_i)\right]$. As before, when H_{pm} is non-Hermitian, the equation of motion above is non-standard.

To proceed in the proof, we use Supplementary Eq. (46) to find that all operators $\hat{O}_{SR}(t)$ with support on the system and pseudomodes space satisfy the equation of motion

$$\frac{d}{dt}\langle[\hat{O}_{SR}]\rangle_{SR} = i\langle[H_{\text{pm}},\hat{O}_{SR}]\rangle_{SR} + \sum_{i=1}^N\langle[D_i^\dagger(\hat{O}_{SR})]\rangle_{SR} \ , \tag{48}$$

where we defined $\langle\cdot\rangle_{SR} = \text{Tr}_{SR}[\ \cdot\ \rho_{SR}(0)]$ [with $\rho_{SR}(0) = \rho_S\prod_{i=1}^N \rho_{R_i}(0)$] and where $D_i^\dagger[\cdot]$ is the adjoint of the operator $D_i[\cdot]$ with respect to the trace, i.e., $\text{Tr}_{SR}[D_i(\hat{A})\hat{B}] = \text{Tr}_{SR}[\hat{A}D_i^\dagger(\hat{B})]$ for any operator $\hat{A}$, $\hat{B}$ with support on the system and pseudomode Hilbert spaces SR. Specifically, $D_i^\dagger[\rho] = \gamma_i/2\left[2a_i^\dagger\rho a_i - (a_i^\dagger a_i\rho + \rho a_i^\dagger a_i)\right]$. In parallel, from Supplementary Eq. (27), we obtain the following Heisenberg equation of motion

$$\begin{aligned} \frac{d}{dt}\langle[\hat{O}_{SR}]\rangle_{SRE} &= i\langle[H'',\hat{O}_{SR}]\rangle_{SRE} \\ &= i\langle[H_{\text{pm}},\hat{O}_{SR}]\rangle_{SRE} \\ &\quad - \sum_{i,\alpha}\frac{g_{i,\alpha}}{\sqrt{2\omega_{i,\alpha}}}\langle b_{i,\alpha}^\dagger[a_i,\hat{O}_{SR}]\rangle_{SRE} \\ &\quad + \sum_{i,\alpha}\frac{g_{i,\alpha}}{\sqrt{2\omega_{i,\alpha}}}\langle[a_i^\dagger,\hat{O}_{SR}]b_{i,\alpha}\rangle_{SRE} \ , \end{aligned} \tag{49}$$

where $\langle\cdot\rangle_{SRE} = \text{Tr}_{E_1}\cdots\text{Tr}_{E_N}\text{Tr}_{SR}$. To close the equations above, we need to compute the equation of motion $\frac{d}{dt}b_{i,\alpha} = i[H'',b_{i,\alpha}]$ for the operators $b_{i,\alpha}(t)$ of the residual baths. This leads to a result which is equivalent to Supplementary Eq. (37), and which reads

$$\sum_\alpha \frac{g_{i,\alpha}}{\sqrt{2\omega_{i,\alpha}}}\bar{b}_{i,\alpha}(t) = b_i^{\text{in}}(t) + A_i(t) \ , \tag{50}$$

where

$$\begin{aligned} b_i^{\text{in}}(t) &= \mathcal{L}_t^{-1}\left[\sum_\alpha \frac{g_{i,\alpha}b_{i,\alpha}(0)}{\sqrt{2\omega_{i,\alpha}}(s+i\omega_{i,\alpha})}\right] \\ A_i(t) &= \mathcal{L}_t^{-1}\left[\sum_\alpha \frac{g_{i,\alpha}^2\bar{a}_i}{2\omega_{i,\alpha}(s+i\omega_{i,\alpha})}\right] \ . \end{aligned} \tag{51}$$

Now, using the convolution theorem and the identities $\mathcal{L}_t^{-1}[1/(s+i\omega)] = \exp(-i\omega t)$ and $\mathcal{L}_t^{-1}[\bar{a}_i] = a_i(t)$, we

notice that, in the continuum limit, the last term in the previous expression can be written as

$$A_i(t) = \frac{1}{\pi}\mathcal{L}_t^{-1}\left[\int_{-\infty}^{\infty} d\omega \frac{J_i(\omega)}{s+i\omega}\bar{a}_i\right] = \frac{\gamma_i}{2\pi}\int_{-\infty}^{\infty} d\omega \int_0^t dt' a_i(t') e^{-i\omega(t-t')} = \frac{\gamma_i}{2} a_i(t) \ , \tag{52}$$

where we used $\int_{-\infty}^{\infty} d\omega e^{i(t'-t)} = 2\pi\delta(t-t')$ and $\int_0^t dt'\delta(t-t) = 1/2$ (see Eq. 5.3.12 in Ref. [15]).

Using Supplementary Eq. (52) and Supplementary Eq. (50) into Supplementary Eq. (49), allows us to write

$$\frac{d}{dt}\langle \hat{O}_{SR}(t)\rangle_{SRE} = i\langle [H_{\text{pm}}, \hat{O}_{SR}(t)]\rangle_{SRE} - \sum_{i=1}^{N}\frac{\gamma_i}{2}\left[\langle a_i^\dagger(t)[a_i(t), \hat{O}_{SR}(t)]\rangle_{SRE} - \langle [a_i^\dagger(t), \hat{O}_{SR}(t)] a_i(t)\rangle_{SRE}\right] = i\langle [H_{\text{pm}}, \hat{O}_{SR}(t)]\rangle_{SRE} + \sum_{i=1}^{N}\langle D_i^\dagger[\hat{O}_{SR}(t)]\rangle_{SRE} \ , \tag{53}$$

where we assumed the initial state of each mode of the residual environment to be the vacuum state, giving $b_{i,\alpha}(0)\rho_{E_i}(0) = \rho_{E_i}(0) b_{i,\alpha}^\dagger(0) = 0$. We can now notice that Supplementary Eq. (48) and Supplementary Eq. (53), despite referring to different underlying spaces, lead to the very same set of closed equation for operators with support in SR, hence predicting the same physical dynamics in such a space. In the Schrödinger picture this results in

$$\text{Tr}_R[\rho_{\text{pm}}(t)] = \rho_S''(t) = \rho_S(t) \ , \tag{54}$$

where we used Supplementary Eq. (33). This completes our proof.

For completeness, it is also interesting to explicitly show that Supplementary Eq. (46) gives, indeed, the same correlations as in Supplementary Eq. (45). In particular, we want to compute the correlations for the "free" pseudomodes, i.e.,

$$C_{\text{pm}}(t) = \text{Tr}_R\left[F(t)F(0)\rho_R(0)\right] \ , \tag{55}$$

where $F(t) = \exp\left(L_R^\dagger t\right)[F(0)]$, with $L_R[\,\cdot\,] = -i[\sum_i \Omega_i a_i^\dagger a_i, \ \cdot\ ] + \sum_i D_i[\ \cdot\ ]$, and where $F(0) = \sum_i \tilde{X}_i^a = \sum_i \lambda_i/\sqrt{2\Omega_i}(a_i^\dagger + a_i)$. We further defined $\rho_R(0) = \prod_i \rho_{R_i}(0)$, where $\rho_{R_i}(0)$ is the initial state of each pseudomodes (which, as before, we assume to be the vacuum state). From its definition, we note that $F(t) = \sum_i \lambda_i/\sqrt{2\Omega_i}\{a_i^\dagger(t) + a_i(t)\}$, where $a_i^\dagger(t) + a_i(t) = \exp\left(L_R^\dagger t\right)[a_i^\dagger + a_i]$. The operator $a_i^\dagger(t) + a_i(t)$ can be found solving the coupled differential equation [to be compared with Supplementary Eq. (43)],

$$\begin{aligned}
\frac{d}{dt}\{a_i^\dagger(t) + a_i(t)\} &= L_R^\dagger\left[a_i^\dagger(t) + a_i(t)\right] \\
&= i\Omega_i\{a_i^\dagger(t) - a_i(t)\} \\
&\quad - \frac{\gamma_i}{2}\{a_i^\dagger(t) + a_i(t)\} \\
\frac{d}{dt}\{a_i^\dagger(t) - a_i(t)\} &= L_R^\dagger\left[a_i^\dagger(t) - a_i(t)\right] \\
&= i\Omega_i\{a_i^\dagger(t) + a_i(t)\} \\
&\quad - \frac{\gamma_i}{2}\{a_i^\dagger(t) - a_i(t)\},
\end{aligned} \tag{56}$$

whose solution can be inserted into Supplementary Eq. (55) to obtain

$$C_{\text{pm}}(t) = C(t) \ , \tag{57}$$

as required.

Supplementary Note 7: Modeling the absence of Matsubara correlations in the generalized pseudomode model

In this section we apply the previous analysis to the case in which the full correlation function in Eq. (2) in the main text is approximated as

$$C(t) \to C_0(t) = \frac{\lambda^2}{2\Omega}\exp\left(-i\Omega t\right)\exp\left(-\frac{\gamma}{2}t\right) \ , \tag{58}$$

i.e., we completely neglect the Matsubara correlations in Eq. (5) from the main text in the zero temperature limit. From Supplementary Eq. (28) we find that this corresponds to an open quantum system in which a single pseudomode (with annihilation operator a) mediates the interaction between the system and the residual environment (with modes associated to annihilation operators

b_α and frequency ω_α) as

$$H_{\rm Mats} = H_S + \sigma_z \frac{\lambda}{\sqrt{2\Omega}}(a + a^\dagger) + \Omega a^\dagger a + \sum_\alpha \omega_\alpha b_\alpha^\dagger b_\alpha + \sum_\alpha \frac{g_\alpha}{\sqrt{2\Omega}\sqrt{2\omega_\alpha}} \left(b_\alpha^\dagger a - a^\dagger b_\alpha\right) . \quad (59)$$

As described in Supplementary Eq. (34), the coupling g_α to the residual environment are determined, in the continuum limit, by the spectral density

$$J_{\rm Mats}(\omega) = \gamma\Omega \ . \quad (60)$$

Note that the apparent additional 2Ω factor in the equation above with respect to Supplementary Eq. (34) just reflects a different definition of the residual couplings in Supplementary Eq. (59) with respect to Supplementary Eq. (28). Alternatively, from the results in the previous section, we also find that the system dynamics can be found by solving

$$\frac{d}{dt}\rho_{\rm eff} = -i[H_{\rm eff}, \rho_{\rm Mats}] + D_{\rm Mats}[\rho_{\rm Mats}] \ , \quad (61)$$

where

$$H_{\rm eff} = H_S + \sigma_z \frac{\lambda}{\sqrt{2\Omega}}(a + a^\dagger) + \Omega a^\dagger a \quad (62)$$

$$D_{\rm Mats}[\rho_{\rm Mats}] = \frac{\gamma}{2}\left(2a\rho_{\rm Mats}a^\dagger - a^\dagger a \rho_{\rm Mats} - \rho_{\rm Mats} a^\dagger a\right) ,$$

and tracing out the pseudomode from $\rho_{\rm Mats}$. The equation of motion in Supplementary Eq. (61) describes the effect of neglecting the Matsubara correlations which are needed to model the correct equilibrium and non-Markovian physics. Consistently, the Lindblad operator in Supplementary Eq. (62) does not describe a residual bath at thermal equilibrium as it does not leave the eigenstates of the system-pseudomode Hamiltonian $H_{\rm eff}$ invariant. This can lead to the possibility of peculiar effects, such as ground state decay and, in general, to a constant dissipation of energy in the steady state. Interestingly, it has been shown (see for a brief overview) that a modified version of the model in Supplementary Eq. (59) can be derived from mapping the original environment into a single "reaction coordinate" and a residual (perturbative) environment. The differences between the two models can be intuitively ascribed to performing a rotating-wave and Markov approximations (in the coupling with the residual bath). Within the perturbative limits for the coupling to the residual environment, the reaction-coordinate model leads to master equations[3,4,16] which improve on Supplementary Eq. (61) to correctly describe the equilibrium and Markovian physics of the original spin-boson model.

Supplementary Note 8: Complete positivity

The pseudomodes mapping presented in the previous section requires the original correlation function to take (or to be approximated by) the functional form given in Supplementary Eq. (35) in terms of a linear combination of decaying exponentials. In the example we focus on in this work, we found that to describe the Matsubara contribution to the correlation, some of the weights λ_i in Supplementary Eq. (35) have to be imaginary. In this case, the effective reduced dynamics of the system $\rho''_S(t)$ might lack fundamental properties like complete positivity. This can be seen by the fact that $\rho''_S(t)$ is obtained by tracing the pseudomodes and their residual environments from the full dynamics $\rho''(t)$ described by the pseudo-Schrödinger equation in Supplementary Eq. (31).

It is useful to consider what further constraints might be imposed on the functional form given in Supplementary Eq. (35) so that the reduced dynamics of the system is completely positive. To achieve this goal, we will follow the same strategy used in the pseudomode proof in the previous section. Namely, we define a bath $B_{\rm CP}$ supporting a specific operator $\hat{X}_{CP}$ coupling to the system such that the full dynamics of system+bath is unitary. In turn, this implies the reduced system dynamics will be completely positive (up to other sources of numerical error which might invalidate the correspondence between the pseudomode model and the full unitary dynamics, see the next subsection). For this reason, we want to study the following relation

$$C_{\rm CP}(t) = C(t)\delta(\Psi) \ , \quad (63)$$

where, slightly generalizing the expression in Supplementary Eq. (35), we write

$$\begin{aligned} C(t) &= \sum_{i=1}^N \tilde{\lambda}_i e^{-(i\Omega_i + \Gamma_i)t} \quad \text{for } t \geq 0 \\ & \sum_{i=1}^N \tilde{\lambda}_i e^{-(i\Omega_i - \Gamma_i)t} \quad \text{for } t < 0 \quad (64) \\ &= \frac{1}{\pi}\int_{-\infty}^{\infty} d\omega \sum_{i=1}^N \Gamma_i \tilde{\lambda}_i \frac{e^{-i\omega t}}{(\omega - \Omega_i)^2 + \Gamma_i^2} \ , \end{aligned}$$

and where $\Psi = \Psi(\{\tilde{\lambda}_i, \Omega_i, \Gamma_i\})$ is a constraint on the parameters of the correlation function needed to allow the identity in Supplementary Eq. (63). In fact, without such a constraint, this equation could not be, in general, satisfied as the bath $B_{\rm CP}$ generates a completely positive reduced dynamics. Finding the explicit expression for Ψ is the goal of this section. In turn, completion of this goal allows to further constraint the parameters defining the correlation in Supplementary Eq. (63), such that the reduced dynamics is formally completely positive. We also defined

$$C_{\rm CP} = \langle \hat{X}_{\rm CP}(t)\hat{X}_{\rm CP}(0)\rangle_{B_{\rm CP}} \ , \quad (65)$$

and

$$\hat{X}_{\rm CP} = \sum \frac{g_i^2}{\sqrt{2|\omega_k|}}(b_k + b_k^\dagger) \ , \quad (66)$$

where the oscillators b_k of the bath are described by the free Hamiltonian $H_{B\mathrm{CP}} = \sum_k \omega_k b_k^\dagger b_k$ for both positive and negative energies. Assuming the kth oscillator to be in the initial vacuum state $|0\rangle_k$, from Supplementary Eq. (65) we then find

$$C_{\mathrm{CP}}(t) = \sum_k \frac{g_k^2}{2|\omega_k|} e^{-i\omega_k t} \equiv \frac{1}{\pi} \int_{-\infty}^{\infty} d\omega J_{\mathrm{CP}}(\omega) e^{-i\omega t} \ , \tag{67}$$

in terms of a “spectral density” (or spectrum) defined for both positive and negative energies as

$$J_{\mathrm{CP}}(\omega) = \pi \sum_k \frac{g_k^2}{2|\omega_k|} \delta(\omega - \omega_k) \ . \tag{68}$$

We now note that the only restriction to the correlation $C_{\mathrm{CP}}(t)$ derives from the fact that the spectral density which defines it satisfies $J(\omega) \geq 0$ as it can be seen from Supplementary Eq. (68). Furthermore, we note that, here, we do not consider any possible issues related to the presence of negative frequencies in the bath. In fact, we are here only concerned about the fact that the full model in which the system interacts with the bath B_{CP} is unitary and, as a consequence, it gives rise to a reduced system dynamics which is completely positive. By inserting the expression in Supplementary Eqs. (64) and (67) into Supplementary Eq. (63) we find, by direct comparison that

$$\sum_{i=1}^{N} \frac{\Gamma_i \tilde{\lambda}_i}{(\omega - \Omega_i)^2 + \Gamma_i^2} = J_{\mathrm{CP}}(\omega) \geq 0 \ . \tag{69}$$

Formally, this implies that

$$\Psi = 1 - \theta \left(\sum_{i=1}^{N} \frac{\Gamma_i \tilde{\lambda}_i}{(\omega - \Omega_i)^2 + \Gamma_i^2} \right) \ , \tag{70}$$

completing the goal of this section. This constraint is a sufficient condition for the original ansatz on the correlation function $C(t)$ to describe a completely positive reduced dynamics for the system.

Truncating the pseudomode Hilbert space

As mentioned throughout the article, the influence of an environment on the system's dynamics is encoded in the correlation functions of the system-bath coupling operator. For this reason, in order to build a pseudomode model which accurately reproduces this dynamics, an equivalence for the full set of correlation functions of the two models is needed. However, when performing numerical simulations of such an equivalent Pseudomode model, it is very important to be aware of approximations that might interfere with these equivalencies.

Specifically, in our proof it is important that both the original bath and the pseudomode simulcra can be described by a Gaussian process in which the two-time correlation functions completely determines the higher order ones. We can then distinguish different classes of numerical errors in simulating the Pseudomode model. Some errors might affect the two-time correlation function while preserving Gaussianity. This is the case for the fit errors considered in this section. While affecting the ability to perfectly reproduce the correct system's dynamics, the Gaussianity condition still allows us to justify fundamental properties, such as complete positivity.

However, it is also possible to have numerical errors which break the Gaussianity condition. A simple example is provided by the unavoidable truncation of the pseudomodes Hilbert space in the numerical implementation of the pseudomode equation of motion. If this truncation is too small, it induces an effective non-linearity which essentially breaks the Gaussianity of the pseudo-mode free evolution. Specifically, since the pseudomode model is described by non-unitary dynamics, these errors are can induce a non-positive reduced system's dynamics, even if the constraints in Supplementary Eq. (63) are satisfied. This is a very different situation from the complete positivity one sees in a standard Lindblad master equation, where insufficient truncation does not affect the positivity condition, so we emphasize that it is important to consider a sufficiently large cut-off for the pseudomode's Hilbert space while performing numerical simulations.

Matsubara fitting example

Let us consider, as an example, the case in which the correlation $C(t)$ takes the form given in Eq. (14) of the main text, i.e.,

$$C(t) = \frac{\lambda^2}{2\Omega} e^{-(i\Omega + \Gamma)t} + c_1 e^{-\mu_1 t} + c_2 e^{-\mu_2 t} \ , \tag{71}$$

at zero temperature. From Supplementary Eq. (69) we need to impose

$$\frac{\Gamma \lambda^2}{2\Omega} \frac{1}{(\omega - \Omega)^2 + \Gamma^2} + \frac{\mu_1 c_1}{\omega^2 + \mu_1^2} + \frac{\mu_2 c_2}{\omega^2 + \mu_2^2} \geq 0 \ , \tag{72}$$

to ensure complete positivity. This expression can be used directly as a cost constraint across a finite frequency range in fitting the parameters c_1, c_2, μ_1, and μ_2. It can also, in principle, be analyzed analytically. To do this, we note that the coefficients c_1 and c_2 are negative so that we can write

$$\frac{\Gamma \lambda^2}{2\Omega} \frac{1}{(\omega - \Omega)^2 + \Gamma^2} \geq \frac{\mu_1 |c_1|}{\omega^2 + \mu_1^2} + \frac{\mu_2 |c_2|}{\omega^2 + \mu_2^2} \ . \tag{73}$$

Now, since

$$\frac{\mu_1 |c_1|}{\omega^2 + \mu_1^2} + \frac{\mu_2 |c_2|}{\omega^2 + \mu_2^2} \leq \frac{\mu_1 |c_1|}{\omega^2 + \mu^2} + \frac{\mu_2 |c_2|}{\omega^2 + \mu^2} \ , \tag{74}$$

where $\mu = \min(\mu_1, \mu_2)$, we can obtain a more restrictive constraint on the parameters by solving

$$\frac{\Gamma\lambda^2}{2\Omega}\frac{1}{(\omega-\Omega)^2+\Gamma^2} \geq \frac{\mu_1|c_1|+\mu_2|c_2|}{\omega^2+\mu^2} \ , \qquad (75)$$

or, equivalently,

$$(A-B)\omega^2 + 2\Omega B\omega + A\mu^2 - B(\Omega^2+\Gamma^2) \geq 0 \ , \qquad (76)$$

where $A = \Gamma\lambda^2/2\Omega$ and $B = \mu_1|c_1| + \mu_2|c_2|$. By imposing that the corresponding parabola is always above or equal to zero we need the coefficient in front of ω^2 to be positive, i.e., we need

$$A/B > 1 \ . \qquad (77)$$

We then impose the determinant of the previous equation to be non-positive, i.e.

$$4\Omega^2 B^2 - 4(A-B)[A\mu^2 - B(\Omega^2+\Gamma^2)] \ \leq \ 0 \ , \qquad (78)$$

which can be recasted as

$$(A/B)^2\mu^2 - (A/B)(\mu^2+\Omega^2+\Gamma^2) + \Gamma^2 \geq 0 \ , \qquad (79)$$

which imposes

$$A/B \leq \kappa_- \quad \text{or} \quad A/B \geq \kappa_+ \ , \qquad (80)$$

where

$$\kappa_\pm = \frac{\mu^2 + (\Omega^2+\Gamma^2) \pm \sqrt{[(\Omega^2+\Gamma^2)-\mu^2]^2 + 4\Omega^2\mu^2}}{2\mu^2} = 1 + \frac{(\Omega^2+\Gamma^2) - \mu^2 \pm \sqrt{[(\Omega^2+\Gamma^2)-\mu^2]^2 + 4\Omega^2\mu^2}}{2\mu^2} \ , \qquad (81)$$

where we used the fact that $(\mu^2+\Omega^2+\Gamma^2)^2 - 4\mu^2\Gamma^2 = (\Omega^2+\Gamma^2-\mu^2)^2 + 4\Omega^2\mu^2$. We now note that (assuming $\Omega, \mu \neq 0$)

$$\sqrt{[(\Omega^2+\Gamma^2)-\mu^2]^2 + 4\Omega^2\mu^2} > (\Omega^2+\Gamma^2) - \mu^2 \ , \qquad (82)$$

so that the second term in the expression for $\kappa_\pm$ is positive (negative) for κ_+ (κ_-). In turn, this implies that

$$\kappa_+ > 1 \ , \qquad \kappa_- < 1 \ . \qquad (83)$$

By using this condition, the constraints in Supplementary Eqs. (77) and (80) are equivalent to just the single condition

$$A/B > \kappa_+ \ , \qquad (84)$$

which, explicitly, reads

$$\frac{\Gamma\lambda^2}{2\Omega(\mu_1|c_1|+\mu_2|c_2|)} > 1 + \frac{(\Omega^2+\Gamma^2) - \mu^2 + \sqrt{[(\Omega^2+\Gamma^2)-\mu^2]^2 + 4\Omega^2\mu^2}}{2\mu^2} \ . \qquad (85)$$

This requirement is then a sufficient condition for the completely positivity of the system's dynamics (again, up to other sources of error, as noted in the previous section). We note that the whole procedure immediately adapts to the case in which an arbitrary number of exponentials are used to fit the Matsubara part of the correlations by simply redefining the parameters μ and B above.

We also recall that the condition in Supplementary Eq. (76), and hence the final result in Supplementary Eqs. (84) and (85), is stricter than the requirement in Supplementary Eq. (72) which is more minimal but also more difficult to implement or check. We also note that it could be interesting to analyze milder conditions in which Supplementary Eq. (76), and hence positivity, is guaranteed only within a certain frequency range. For example, in the case $A > B$, this can be done by allowing a positive determinant in Supplementary Eq. (76). In this case, the range of frequencies in which positivity is guaranteed would be around infinity. Interestingly, the physically more relevant case in which the range of frequencies is around some finite value would arise when $A < B$.

[1] A. Garg, J. N. Onuchic, and V. Ambegaokar, "Effect of friction on electron transfer in biomolecules," J. Chem. Phys. **83**, 4491–4503 (1985).

[2] R. Martinazzo, B. Vacchini, K. H. Hughes, and I. Burghardt, "Universal Markovian reduction of Brownian particle dynamics," J. Chem. Phys. **134**, 011101 (2011).

[3] J. Iles-Smith, N. Lambert, and A. Nazir, "Environmental dynamics, correlations, and the emergence of noncanonical equilibrium states in open quantum systems," Phys. Rev. A **90**, 032114 (2014).

[4] P. Strasberg, G. Schaller, N. Lambert, and T. Brandes, "Nonequilibrium thermodynamics in the strong coupling and non-Markovian regime based on a reaction coordinate mapping," New J. Phys. **18**, 073007 (2016).

[5] G.-L. Ingold, "Path integrals and their application to dissipative quantum systems," in *Coherent Evolution in Noisy Environments* (Springer, 2002) pp. 1–53.

[6] R. Loudon, *The Quantum Theory of Light* (Oxford Science Publ., 1992).

[7] L. Zhu, H. Liu, W. Xie, and Q. Shi, "Explicit system-bath correlation calculated using the hierarchical equations of motion method," J. Chem. Phys. **137**, 194106 (2012).

[8] L. Song and Q. Shi, "Hierarchical equations of motion method applied to nonequilibrium heat transport in model molecular junctions: Transient heat current and high-order moments of the current operator," Phys. Rev. B **95**, 064308 (2017).

[9] C. Schinabeck, R. Härtle, and M. Thoss, "Hierarchical quantum master equation approach to electronic-vibrational coupling in nonequilibrium transport through nanosystems: Reservoir formulation and application to vibrational instabilities," Phys. Rev. B **97**, 235429 (2018).

[10] F. Mascherpa, A. Smirne, S. F. Huelga, and M. B. Plenio, "Open systems with error bounds: Spin-boson model with spectral density variations," Phys. Rev. Lett. **118**, 100401 (2017).

[11] H.-S. Goan, C.-C. Jian, and P.-W. Chen, "Non-Markovian finite-temperature two-time correlation functions of system operators of a pure-dephasing model," Phys. Rev. A **82**, 012111 (2010).

[12] B. M. Garraway, "Nonperturbative decay of an atomic system in a cavity," Phys. Rev. A **55**, 2290–2303 (1997).

[13] D. Tamascelli, A. Smirne, S. F. Huelga, and M. B. Plenio, "Nonperturbative treatment of non-Markovian dynamics of open quantum systems," Phys. Rev. Lett. **120**, 030402 (2018).

[14] A. Lemmer, C. Cormick, D. Tamascelli, T. Schaetz, S. F. Huelga, and M. B. Plenio, "A trapped-ion simulator for spin-boson models with structured environments," New J. Phys. **20**, 073002 (2018).

[15] C. Gardiner and P. Zoller, *Quantum noise: a handbook of Markovian and non-Markovian quantum stochastic methods with applications to quantum optics*, Vol. 56 (Springer Science, 2004).

[16] J. Iles-Smith, A. G. Dijkstra, N. Lambert, and A. Nazir, "Energy transfer in structured and unstructured environments: master equations beyond the Born-Markov approximations," J. Chem. Phys. **144**, 044110 (2016).
